# Editorial: Biologics for airway diseases: from bench to bedside

**DOI:** 10.3389/falgy.2026.1840104

**Published:** 2026-04-10

**Authors:** Andreas M. Matthaiou, Nikoleta Bizymi, Dina Visca, Lena Uller, Nikolaos G. Papadopoulos, Alexander G. Mathioudakis

**Affiliations:** 1 5th Department of Respiratory Medicine, Sotiria Thoracic Diseases General Hospital of Athens, Athens, Greece; 2 Department of Medicine and Cardiopulmonary Rehabilitation, Istituti Clinici Scientifici Maugeri IRCCS, Tradate, Italy; 3 Department of Medicine and Surgery, University of Insubria, Varese, Italy; 4 Unit of Respiratory Immunopharmacology, Department of Experimental Medical Science, Lund University, Lund, Sweden; 5 Allergy Department, 2nd Pediatric Clinic, National and Kapodistrian University of Athens, Athens, Greece; 6 Lydia Becker Institute, The University of Manchester, Manchester, United Kingdom; 7 Division of Immunology, Immunity to Infection and Respiratory Medicine, Faculty of Biology, Medicine and Health, The University of Mancester, Manchester, United Kingdom; 8 North West Lung Centre, Wythenshawe Hospital, Manchester University NHS Foundation Trust, Manchester Academic Health Science Centre, Manchester, United Kingdom

**Keywords:** airway diseases, asthma, biologics, chronic rhinosinusitis with nasal polyposis (CRS wNP), COPD

Over the past decade, biologic therapies have transformed the management and outcomes of type 2 (T2) severe asthma, with more recent evidence and regulatory approvals suggesting similar benefits in selected patients with chronic obstructive pulmonary disease. Targeting upstream mediators such as epithelial alarmins has begun to extend these approaches beyond classical T2-high disease (1). However, important questions remain unresolved, including incomplete response and loss of efficacy over time in a subset of patients. At the same time, a substantial proportion of patients with airway diseases remain poorly characterised and inadequately treated (2). Recent advances, including the demonstration of the dipeptidyl peptidase 1 inhibitor brensocatib's efficacy in bronchiectasis, challenge the traditional paradigm that infection predisposition should be managed primarily with antimicrobial strategies, instead highlighting the potential of targeting host inflammatory pathways to modify disease risk and outcomes (3). Together, these advances signal a shift from empirical treatment towards mechanism-based therapy in airway diseases.

Notably, much of this progress has been built on a relatively narrow set of biomarkers reflecting a single dominant trait-T2 inflammation. While these have enabled major advances in targeted therapy, they capture only part of the biological complexity and heterogeneity underlying airway diseases. This marked heterogeneity contributes to variable clinical outcomes and highlights the limitations of “one-size-fits-all” approaches (4). Moving forward, a step change in disease characterisation is required, integrating multi-omics data with deep and broad clinical phenotyping to better define diverse endotypes and uncover novel treatable traits, including mechanisms related to airflow limitation, mucus dysfunction, environmental exposures, and comorbidities. Expanding this framework will be essential to advance precision medicine across the full spectrum of airway diseases (5, 6).

Within this evolving landscape, this Research Topic brings together studies spanning the full translational spectrum, from fundamental mechanistic insights to clinical application, reflecting the rapid evolution of biologics in airway diseases. Studies investigating immunomodulatory therapies in both *in vitro* and *in vivo* models of asthma have revealed their multidimensional effects on disease biopathology beyond clinical benefits. The enhancement of antiviral responses and the mitigation of viral-induced inflammatory cascade in asthma upon treatment with macrolides and biologics was outlined in the two studies by Ghanizada et al. and Pesic et al., respectively. The first group showed that the treatment of cultured bronchial epithelial cells from asthma patients, including eosinophilic, non-eosinophilic, and non-atopic phenotypes, with azithromycin led to significantly enhanced release of interferon (IFN) *β* and IFN-*λ* as a response to *in vitro* rhinoviral infection. These findings suggest a potential role for add-on macrolide therapy, particularly in asthma patients who do not respond adequately to biological treatments. Pesic and colleagues reported that the presence of anti-interleukin (IL)-4 receptor *α* during *in vitro* rhinovirus infection of cultured bronchial epithelial cells from asthma patients reduced the production of the eosinophil chemoattractant (C-C motif) ligand 26 and thymic stromal lymphopoietin, two potent mediators of T2 immunity. Furthermore, Wang et al. investigated the effects of long-term administration of bacterial lysates in a murine model of allergic asthma, demonstrating alleviated symptoms, reduced IL-4, IL-5, IL-13, and neurokinins, elevated IFN-*γ*, IL-10, regulatory T cells, and CD103^+^ dendritic cells, as well as higher abundance of *Lactobacillus spp.*, further suggesting lung–gut axis as a promising therapeutic target in asthma. Together, these translational studies highlight the capacity of immunomodulatory therapies to enhance antiviral responses and modulate airway inflammation across asthma phenotypes.

Biologics have revolutionised asthma management, although their benefits are occasionally limited by inadequate response in a subset of patients, while they also require parenteral administration at frequent time intervals. Depemokimab is the first ultra-long-acting monoclonal antibody targeting IL-5. The efficacy of this novel biologic in patients with T2-high asthma and comorbid chronic rhinosinusitis with nasal polyps was shown by Heffler et al. using pooled data from the SWIFT-1 and SWIFT-2, two phase III, multicentre, randomised, double-blind, placebo-controlled trials. The authors reported a significant reduction in annual exacerbation rates and improvement in asthma symptom control by treating patients two times yearly, indicating the efficacy of depemokimab in patients with unified airway involvement. On the other hand, sputum neutrophilia and its impact on the effect of biologics in severe asthma was investigated by Pignatti et al. After 12 months of biologic therapy, the patients with neutrophilic airway inflammation did not show significant improvement in their pulmonary function, compared to those with eosinophilic or mixed granulocytic types, indicating airway neutrophilia as a negative predictor of the efficacy of biologics targeting T2-high inflammation. These findings highlight both the promise of novel biologics and the critical role of disease heterogeneity in shaping treatment response.

In addition to original research, this Research Topic is enriched by review and perspective contributions that delineate current challenges in asthma management, propose systematic approaches to guide biological therapy, and underscore promising directions for future investigation. The importance of optimal management of patients with severe asthma was summarised by Yasui, highlighting the limitations across all steps from diagnosis through long-term follow-up. According to the author's perspective, the four major pillars of future research goals include: i) the investigation of novel biomarkers incorporating multi-omics approaches and microRNA data; ii) the identification of independent predictors through large-scale studies; iii) the use of registries and existing datasets to identify associations, facilitate prospective studies, and promote personalised medicine; and iv) patient-centred care, including questionnaires, surveys, and shared decision-making on the biologic choice and management plan. On the same note, Panaitescu et al. proposed a structured strategy for the selection of biologics in airway diseases. Firstly, they introduced a multidimensional framework for allergic disease stratification, encompassing clinical, biological (i.e., phenotypes reflecting observable biological traits), environmental (i.e., regiotypes reflecting environmental and geographical influences), and therapeutic dimensions (i.e., pharmacotypes reflecting efficacy of different agents and theratypes referring to prediction of therapeutic responsiveness). Secondly, they presented two stepwise algorithms guiding biologic therapy in severe asthma and chronic rhinosinusitis with nasal polyps based on their stratified endotypes. Finally, Chen et al. stressed the need for next-generation therapeutic modalities due to the inherent drawbacks of the currently available biologics, particularly the long administration period and the low rates of disease remission. The authors argued on the promising role of cell therapies for severe asthma, such as reprogrammed T2 helper T cells or engineered chimeric antigen receptor natural killer cells. These perspectives underscore the need for integrated, biomarker-informed strategies to advance precision medicine in airway diseases.

We are still in the midst of a therapeutic revolution in airway diseases. While major advances have transformed the management of T2 inflammation, important unmet needs persist, particularly in non-T2 disease and other under-recognised treatable traits. Progress will depend on stronger integration across basic, translational, and clinical research, and on sustained collaboration across disciplines to drive more precise and equitable care.
